# Effects of sub-lethal doses of fentanyl on vital physiologic functions and withdrawal-like behaviors in adult goats

**DOI:** 10.3389/fphys.2023.1277601

**Published:** 2023-10-11

**Authors:** Suzanne E. Neumueller, Nicole Buiter, Grace Hilbert, Kirstyn Grams, Reiauna Taylor, John Desalvo, Grace L. Hodges, Madeline M. Hodges, Lawrence G. Pan, Stephen J. Lewis, Hubert V. Forster, Matthew R. Hodges

**Affiliations:** ^1^ Department of Physiology, Medical College of Wisconsin, Milwaukee, WI, United States; ^2^ Department of Physical Therapy, Marquette University, Milwaukee, WI, United States; ^3^ Departments of Pediatrics and Pharmacology, Case Western Reserve University, Cleveland, OH, United States; ^4^ Zablocki Veterans Affairs Medical Center, Milwaukee, WI, United States; ^5^ Neuroscience Research Center, Medical College of Wisconsin, Milwaukee, WI, United States

**Keywords:** OIRD, control of breathing, fentanyl, ventilation, EMG

## Abstract

Synthetic opioids like fentanyl have improved the standard of care for many patients in the clinical setting, but their abuse leads to tens of thousands of overdose deaths annually. The current opioid epidemic underscores a critical need for insights into the physiological effects of fentanyl on vital functions. High doses of opioids in small mammals cause opioid-induced respiratory depression (OIRD) leading to hypoventilation, hypoxemia, and hypercapnia. In addition, opioids can also increase the alveolar to arterial oxygen (A-a) gradient and airway dysfunction. However, little is known about the physiologic effects of sub-lethal doses of opioids in large mammals. Here we report the effects of a sub-lethal dose range of fentanyl (25–125 μg/kg; IV) on vital physiologic functions over 90 min (min) and withdrawal-like behaviors over the subsequent 4 h (h) in adult female goats (*n* = 13). Fentanyl induced decreases in breathing frequency in the first few min post-injection, but then led to a sustained increase in tidal volume, total ventilation, and blood pressure with a reduced heart rate for ≥90 min. These ventilatory changes resulted in time-dependent arterial hypocapnia and hypoxemia and an increased alveolar to arterial oxygen gradient ∼30 min post-injection indicative of impaired gas exchange in the lung. The predominant effects of fentanyl on breathing were stimulatory, underscored by an increased rate of rise of the diaphragm muscle activity and increased activation of upper airway, intercostal and abdominal muscles. Beginning 90 min post-injection we also quantified withdrawal-like behaviors over 4 h, demonstrating dose- and time-dependent increases in locomotor, biting, itching, and pawing behaviors. We conclude that fentanyl at sublethal doses induces multiple physiologic and behavior changes that emerge along different time courses suggesting multiple independent mechanisms underlying effects of opioids.

## Introduction

The development of synthetic opioids like fentanyl has improved the standard of care for patients in the clinical setting. However, the use and abuse of fentanyl has skyrocketed leading to tens of thousands of overdose deaths annually, which has stimulated studies to gain insight into the life-threating physiologic effects of opioids. Current data suggest fentanyl impairs ventilatory function by at least three mechanisms to elicit life-threatening hypoxemia. One major effect is opioid depression of CNS-driven ventilatory output termed Opioid-induced Respiratory Depression (OIRD). The neural circuitry that generates respiratory rhythm and motor patterns largely reside in the brainstem and includes regions like the pre-Bötzinger Complex (pre-BötC), the post-inspiratory complex (PiCO), and rostral pontine nuclei (Smith et al., 1991; St John, 1999; Anderson et al., 2016). Several studies indicate neurons in this key respiratory control circuit may be uniquely sensitive to opioid inhibition and underlie hypopnea/hypoventilation under physiologic conditions (Montandon and Horner, 2014; Anderson et al., 2016). A second ventilatory impairment elicited by opioids is skeletal muscle rigidity, including accessory respiratory muscles in the chest wall to create what is clinically known as “wooden chest syndrome” (WCS) (Marty and Desmonts, 1981; Drummond, 1983; Scamman, 1983; Weinger et al., 1988; Neidhart et al., 1989; Weinger and Bednarczyk, 1994; Lui et al., 1995; Bowdle, 1998). Synchronous activation of normally reciprocal respiratory muscles (diaphragm and abdominal muscles, internal and external intercostals) and upper airway muscles results from what appears to be an extreme simultaneous stimulation of respiratory pump and airway muscles (Scamman, 1983; Abrams et al., 1996; Bennett et al., 1997). A third respiratory impairment induced by opioids is pulmonary reductions in oxygen (O_2_) exchange leading to an increase in the alveolar to arterial (A-a) O_2_ gradient (Meyer et al., 2010; Getsy et al., 2022), which may have as great of an effect as apneas to reduce PaO_2_ after opioid administration. High dose opioids impair alveolar-capillary gas exchange presumably due to ventilation-perfusion mismatch within the lung.

Each of the effects of opioids alone or in combination could be life-threatening and may depend upon the timing of their occurrence. Decreases in ventilatory frequency presumably *via* inhibition of respiratory rhythm generation suggests that opioids can be life threatening because of a progressive CNS-mediated frequency decline leading to cessation of diaphragmatic activity and airflow (central apnea; CA). However, cessation of respiratory airflow also occurs even when respiratory rhythm generation is sustained but the airway is closed to induce an obstructive apnea (OA). The mechanism underlying opioid-induced OA is likely part of the WCS phenomenon *via* tonic activation of expiratory airway muscle and/or active vocal cord closure (Torralva and Janowsky, 2019). WCS and OA often emerge in less than 2 min after opioid intravenous (IV) injections, usually last up to 15 min, and presumably could cause death with a more accelerated time course than CA (Streisand et al., 1993). The mechanism(s) and timing of opioid-induced increases in the A-a O_2_ gradient are less clear but have been shown to develop with a slower time course than OIRD (Meyer et al., 2010; Getsy et al., 2022). Withdrawal symptoms manifest for hours after fentanyl use, and include symptoms such as hyperactivity, anxiety, and repetitive behaviors among others (Varshneya et al., 2019; Digranes et al., 2023). Accordingly, there is a need for a better understanding of the timing and magnitude of fentanyl on multiple vital physiological functions and potential withdrawal-like behaviors, particularly in a large animal model and across a dose range of fentanyl that does not cause sustained apnea or death.

Accordingly, herein we studied healthy, intact, freely-behaving adult goats to gain insight into time- and dose-dependent physiological effects and withdrawal-like behaviors following intravenous (IV) administration of fentanyl (2 min) over a range of doses that cause minimal or no OA. This design allowed for the characterization of the temporal pattern of each potential cause of fentanyl-induced hypoxemia. We tested the hypotheses that sublethal doses of opioids will induce both dose- and time-dependent dysfunction in multiple physiologic functions, including breathing frequency suppression (OIRD), increased tonic (or phasic) activation of chest wall and upper airway respiratory muscles (WCS) leading to a delayed or lack of inspiratory flow, arterial hypertension, reduced heart rate, and reductions of reflexes such as sighs and swallows. We further hypothesize that fentanyl will also increase acute withdrawal-like behaviors in adult goats like those seen in small mammals and humans (Varshneya et al., 2019; Digranes et al., 2023). Data from sublethal fentanyl doses will be important to clinicians in decisions regarding use of opioids as an analgesic after surgery and in chronic conditions of persistent pain.

## Methods

All protocols were reviewed and approved by The Medical College of Wisconsin (MCW) Institutional Animal Care and Use Committee (IACUC). This institution has an Animal Welfare Assurance on file with the Office of Laboratory Animal Welfare. The Assurance Number is D16-00064 (A3102-01).

Adult female (>2y) goats (*n* = 13) of various breeds (i.e., Alpine, Toggenburg, Saanen) weighing 49.1 ± 2.9 kg were obtained from a local outbred herd. Goats were reared, transported, housed, and cared for according to USDA regulations. Upon arrival to the lab blood samples were drawn (jugular puncture) for complete blood count panels and pregnancy testing. Deworming conditioning (Ivermectin, 0.2 mg/kg, SQ); Fenbendazole, 5 mg/kg, PO) also occurred upon arrival and was repeated 2 weeks later.

Animal preparation (surgical instrumentation and recovery): As described previously (Feroah et al., 2002; Feroah et al., 2003), the goats underwent aseptic surgery for subcutaneous relocation of the carotid arteries bilaterally (for blood sampling and BP/HR monitoring) and implantation of EMG electrodes into airway (genioglossus and thyropharyngeus) and respiratory pump (diaphragm, intercostal, and transverse abdominus) muscles. Goats were anaesthetized (ketamine; 15 mg/kg, IV) for intubation and mechanically ventilated under 2%–3% isoflurane anesthesia (in 100% O_2_). Post-surgical health assessments (body temperature, food/water intake, surgical incision healing, *etc.*) were completed daily by trained staff, and goats received Banamine or Flunixin Meglumine (2 mg/kg, IM) and Naxcel (4 mg/kg, IM) for 3 and 7 days post-op, respectively. Unilateral carotid catheterization was performed ∼14 days post-op after which Naxcel (4 mg/kg, IM) was administered daily. Indwelling catheters were flushed daily with heparinized saline (0.1% heparin) followed by heparin lock solution.

Study preparation: Studies began ≥2 weeks post-surgery and following a week of training to familiarize the goats to the equipment. Goats were tethered with ropes and a harness in a stanchion and fitted with a custom ventilatory mask and one-way breathing valve adhered to the snout. The inspiratory and the expiratory ports of the valve were each connected to separate hoses and the inspired hose connected to a calibrated pneumotachometer whereas the expiratory hose was connected to a Tissot gasometer for collection of expired gases. An IV catheter was placed and secured unilaterally in the jugular vein daily prior to study (<30 min prior to injections). Muscle electromyographic (EMG) wires were clipped to cables and an arterial catheter line attached to a manifold and pressure transducer to allow for arterial blood sampling and measurement of blood pressure/heart rate. Rectal temperatures were recorded at regular intervals.

**FIGURE 1:**
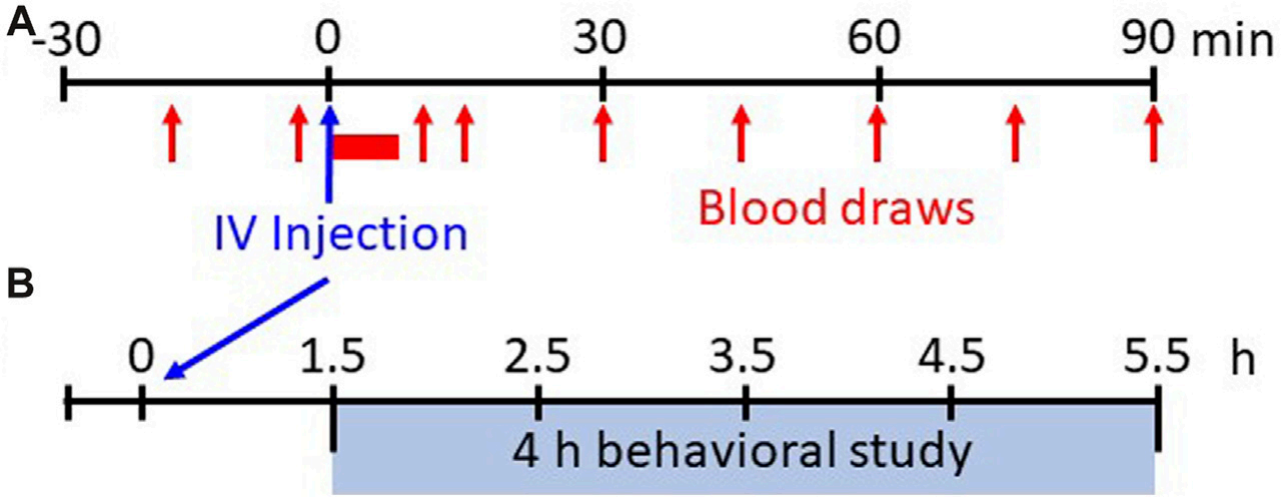
Experimental protocols. Shown in **(A)** is the experimental protocol for physiological measures for 30 min before and up to 90 min after IV injections of saline or various doses of fentanyl. Breathing, blood pressure/heart rate, and EMG activity are measured continuously whereas arterial blood samples are drawn at 15 min intervals during the pre-injection period and beginning 15 min post-injection (serial blood samples taken from 1–5 min post-injection initiation). Shown in **(B)** is the experimental protocol for withdrawal-like behavior monitoring. Following 90 min post-injection the animals are placed in a custom pen wearing a Bluetooth bio-harness and video recordings (HD webcam) are captured for offline analyses of locomotor behaviors beginning 1.5 h–5.5 h (4 h total) post-injection.

Study protocol: Physiologic measurements were recorded for 30 min before and for 90 min following IV injections of saline (0.9% NaCl) or fentanyl (25, 50, 75, 100, or 125 μg/kg; Hospira or West-Ward (50 μg/mL solution). Fentanyl dose order was randomized and distributed over approximately 4–5 weeks (≥48 h between studies). The dose range selected includes clinical intraoperative doses used in humans (20–50 μg/kg) to a level well below doses considered as “high” doses in rodents (<300 μg/kg) (Fujii et al., 2019). Arterial blood was sampled at regular intervals (every 15 min during pre-injection period; every 1 min for up to 5 min post-injection; every 15 min thereafter; Figure 1A). Study equipment (i.e., mask, valve, EMG cables, catheter lines) was removed after completion of the 120 min study and then the goats were fitted with a wearable Bioharness (Biopac Systems Inc, Goleta, CA, United States) and placed in a clear plexiglass pen (3.5′ x 4′ x 6’) equipped with an HD video camera (Logitech Webcam C930e) for recording withdrawal-like behaviors (Figure 1B) during which time the goat did not have access to food or water (4 h). Goats had access to food/water *ad libitum* other than pre-surgical fasting or during studies. After completion of all studies the goats were euthanized with IV ketamine/xylazine (2.5 mL; 24:1) and IV phenytoin/phenobarbital (B-euthanasia, 97.5 mg/kg) and the brains extracted (<10 min), rapidly frozen (2-methylbutane and dry ice) and stored at −80 °C for future studies.

**FIGURE 2:**
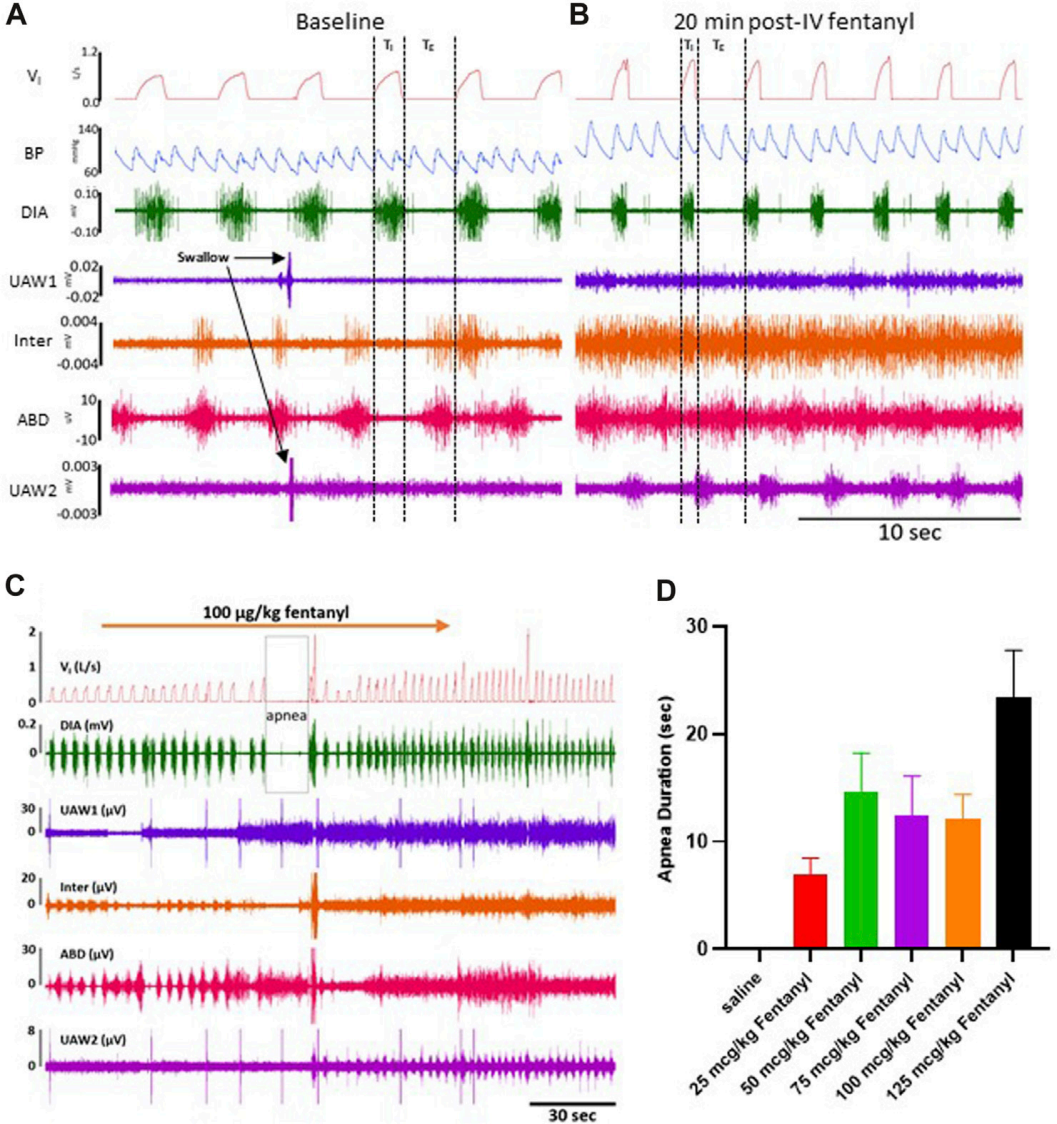
Representative tracing during baseline and 50 μg/kg fentanyl. Inspiratory flow, arterial pulse pressure, and muscle activity is measured during a control period **(A)** followed by an intravenous (IV) injection (2 min) of 50 μg/kg fentanyl. ${\dot{V}}_{I}$ , inspiratory flow; BP, arterial blood pressure; DIA, diaphragm muscle EMG; UAW1, upper airway muscle 1 EMG; Inter, intercostal muscle EMG; ABD, transverse abdominal muscle EMG; UAW2, upper airway muscle 2 EMG. Note that swallows (arrows) were detectable by large bursts of synchronous activity in both upper airway muscles (genioglossus (UAW1) and thyropharyngeus (UAW2)). Note also that 20 min post-injection of 50 μg/kg fentanyl (IV; **(B)** that V_T_ was increased with shortening of T_I_, arterial blood pressure(s) were increased and heart rate was decreased, and there were general increases in upper airway, intercostal and abdominal muscle EMG activities. Shown in **(C)** are representative data of the acute physiological effects of 100 μg/kg fentanyl to elicit a transient apnea. Shown in **(D)** is the mean ± SEM of all apnea durations with a given dose of fentanyl or saline (*n* = 13).

Data Acquisition and Analysis: Powerlab and LabChart software (ADInstruments, Colorado Springs, CO, United States) was used for the continuous recording of inspiratory flow, blood pressure/heart rate, and respiratory muscle (EMG) activity (Figure 2A). A gas analyzer (OxiGraf, Sunnyvale, CA, United States) was utilized for measurement of mixed expired gas composition, i.e., expired O_2_ (F_E_O_2_) and CO_2_ (F_E_CO_2_) collected in a Tissot gasometer (5 min collections) from which oxygen consumption ( $\dot{V}$ O_2_) was calculated. Arterial pH, blood gases, and electrolytes were measured with a PRIME CCS blood gas analyzer (Nova Biomedical, Waltham, MA, United States) and corrected to animal temperatures. After completing blood gas measurements arterial blood samples were centrifuged (2,500 rpm for 15 min) and plasma collected and frozen. During this process we noted a red hue in the plasma despite complete plasma/red cell separation, suggesting that at higher doses we may have notable hemolysis (Supplementary Figure S1A). In a few experiments we tested if dilution of the fentanyl solution (1:1 with saline) would reduce this effect and which was confirmed. Despite the reduction in hemolysis there were no differences between dilute and non-dilute physiological effects including the A-a O_2_ gradient (*n* = 5–6; Supplementary Figure S1B-C3). Blood pressures (mmHg) were measured from a calibrated pressure transducer from which peak (systolic), minimum (diastolic) and mean (1/3 systolic–2/3 diastolic) blood pressure calculations along with heart rate (beats/min) were calculated and averaged over 5–15 min intervals. Care was taken to adjust the height of the transducer when the animal went from standing to laying to remain at heart level. The A-a O_2_ gradient was calculated as P_A_-PaO_2_, where P_A_ = (0.209*(barometric pressure - water vapor pressure) - PaCO_2_) as previously described (Meyer et al., 2010) and averaged over 5–15 min intervals. Bioharness data (vector magnitude; g) was collected in Biopac software (Acqknowledge) and HD video recordings were viewed offline for event counts of biting, rearing, vocalization, pawing, and itching behaviors and averaged over 30 min intervals. Breathing frequency (F_B_; breaths/min), tidal volume (V_T_; L/breath) and ${\dot{V}}_{I}$ ; F_B_*V_T_; L/min) were calculated on a breath-by-breath bases as previously described (Langer et al., 2017a; Langer et al., 2017b; Burgraff et al., 2018) in 1 s intervals and then averaged in 30 s or 5 min bins for 15 min before or 15 min post-injection. Inspiratory (T_I_; sec) and expiratory time (T_E_; sec) was derived from breath-by-breath calculation of inspiratory flow times (T_I_) and subtracting it from total breath cycle time (T_E_). Diaphragmatic (DIA) EMG analyses were conducted by digital filtering, rectification, smoothing (400 ms) and integration of the raw DIA signals to obtain area under the curve (AUC), and using the “peak analysis” function in LabChart to obtain the rate of rise (mV/sec) which were binned in 1 min or 15 min intervals. Similarly, raw genioglossus, thyropharyngeus, intercostal and abdominal EMG signals were digitally filtered (High Pass, 180–190 Hz) and integrated breath-by-breath and averaged over 5 min periods at the end of the baseline and from minutes 10–15, 25–30 and 85–90 post-injection. Swallows (synchronous bursts in genioglossus and thyropharyngeus activity), augmented breaths, and obstructive apneas (OAs) were counted manually offline.

Statistical analyses were completed using One- or Two-way RM ANOVA or Mixed-effects Analysis (if there were missing data) with Dunnett’s or another appropriate *post hoc* test (Time and Dose factors; GraphPad Prism 8 Software), and *p* < 0.05 except wherever noted. All statistical tests (and confidence intervals) used are reported in the figure legends or Results section.

## Results

All fentanyl injections (2 min; IV) caused all goats to lay supine from a standing posture during or shortly after completing the injection of fentanyl while animals given IV saline injections remained standing (*n* = 13). The time from initiation of the injection to laying with 25 μg/kg fentanyl was 3.04 ± 0.39 min which decreased to 1.85 ± 0.22 min (50 μg/kg), 1.59 ± 0.19 min (75 μg/kg), 1.43 ± 0.11 min (100 μg/kg) and 1.58 ± 0.16 min (125 μg/kg) with increasing doses (*p* ≤ 0.014; Supplementary Figure S2A). Once supine, the animals appeared sedated and largely immobilized with the exceptions of voluntary repositioning side to side, grinding their teeth and/or occasional bouts of coughing. Periods of these events were deleted from the recordings before quantitation of physiologic responses. The time animals remained in the laying position was dose-dependent, where they voluntarily stood after 55.2 ± 7.7 min, 70.4 ± 6.3 min, 81.8 ± 4.0 min, 82.7 ± 3.7 min, or 89.3 ± 0.3 min after 25, 50, 75, 100 or 125 μg/kg fentanyl, respectively, where the time to stand was longer for all doses >75 μg/kg compared to 25 μg/kg (*p* ≤ 0.005; Supplementary Figure S2B). It is noteworthy that the time to stand for the higher doses was affected by the removal of instrumentation from the animal at the end of the 90 min experiment, and at times the goats would remain immobilized requiring assistance to be transferred to the behavioral chamber.

Due to their immobilization with fentanyl, we assessed potential changes in protective behaviors (sighs and swallows) along with obstructive apneas (OAs) during baseline conditions and up to 90 min post-injection (Supplementary Figure S2C-E). Swallows occurred frequently during baseline conditions (∼2/min) and continued without change after saline injection but decreased for 90 min post-injection of all doses of fentanyl (*p* < 0.05; Supplementary Figure S2C). Sighs, which occur regularly to reinflate alveoli and prevent atelectasis, did not differ during baseline conditions but generally increased at various timepoints for all doses of fentanyl (*p* < 0.05; Supplementary Figure S2D). OAs (defined as T_E_> 2x baseline) were very infrequent across all conditions and timepoints and were only increased initially after 100 μg/kg fentanyl in the first 30 min after injection (*p* = 0.0096; Supplementary Figure S2E).

The majority of goats responded to fentanyl with transient decreases in F_B_ (*via* increased T_E_) and sustained increases in V_T_ and V_I_ (representative data shown in Figure 2). In addition, we also typically observed increases in tonic activation of the upper airway (UAW), intercostal and abdominal muscles after fentanyl injections (Figure 2). Furthermore, heart rate (HR) decreased and arterial blood pressures were increased with IV fentanyl (Figure 2). However, the effects of fentanyl on breathing frequency (F_B_), tidal volume (V_T_) and minute ventilation ( ${\dot{V}}_{I}$ ) were variable across animals (Supplementary Figure S3). For example, sustained reductions in F_B_ (>-15% for ≥2 min) within the first 15 min post-fentanyl injections (one measure of OIRD) was only noted in 31%–54% of all experiments across all doses. Despite variability in responses, all goats tested for each dose are included in group means to illustrate the overall effects of fentanyl. After completing all doses of fentanyl, we repeated the 50 mg/kg dose and found excellent reproducibility for the frequency and PaCO_2_ responses but there was variation between repeat studies in the ${\dot{V}}_{I}$ and V_T_ responses which were lower in the repeat studies (Supplementary Figure S4). Overall, the effects on F_B_ were transient with the greatest effects occurring within 1–5 min of the initiation of fentanyl injections. Shown in Figure 2C is an example of a transient central apnea (14.8 s) elicited during the injection of 100 μg/kg fentanyl. All apneas (OA, extended T_E_ and post-sigh) were quantified across saline and fentanyl doses (Figure 2D) showing a general trend for increasing apnea length with increasing dose. Most (69%–100%) of the animals exhibited transient apnea (OA, prolonged T_E_ or post-sigh) of varying length during the fentanyl injections (7.0–23.5 s on average across all doses; n = 13; Figures 2C,D).

**FIGURE 3:**
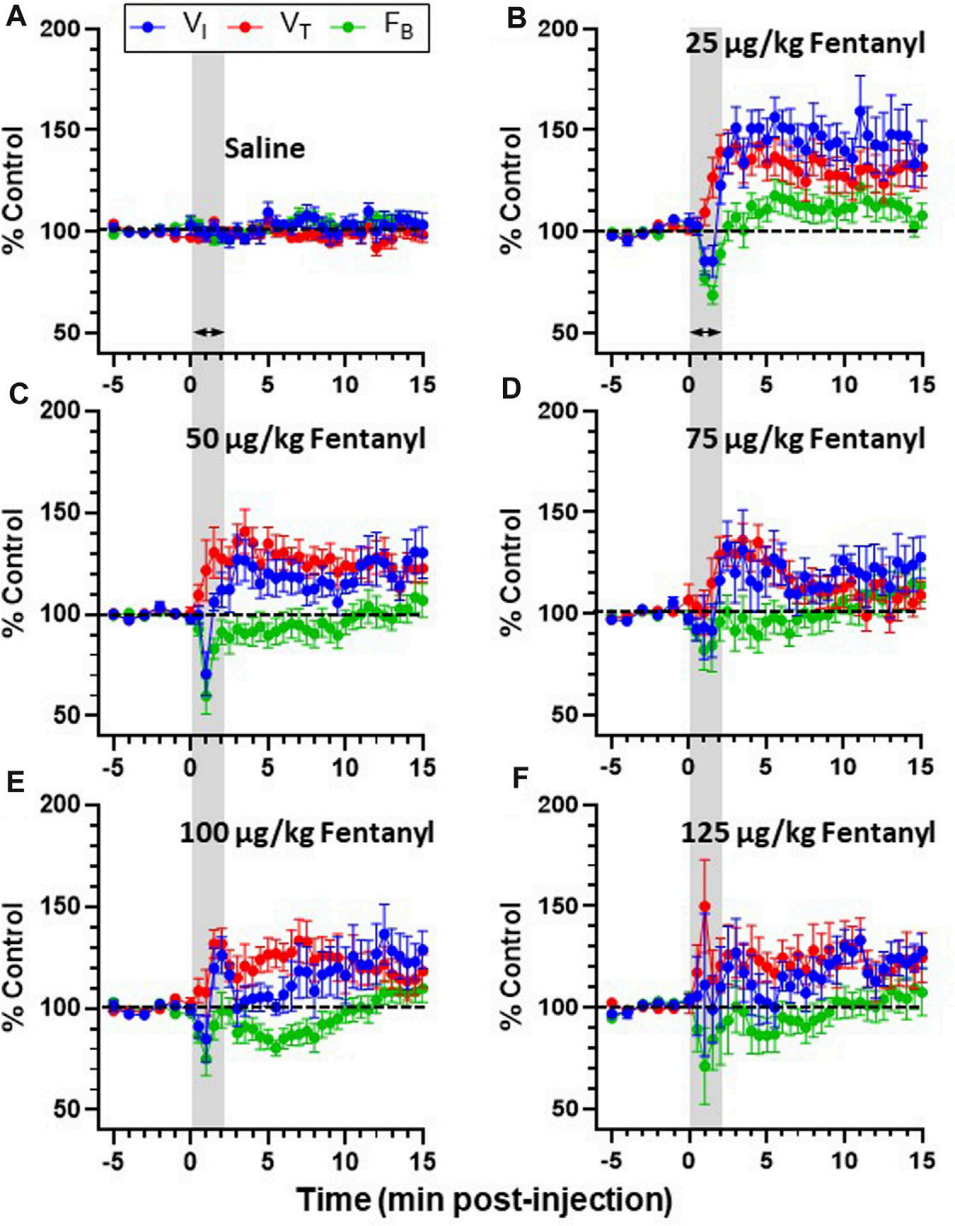
Effects of fentanyl on breathing measures in adult goats. Minute ventilation ( ${\dot{V}}_{I}$ ; L/min), breathing frequency (F_B_; breaths/min) and tidal volume (V_T_; L/breath) expressed as a percentage of control (baseline) from 5 min before and up top 15 min following IV injections of saline (**A**; n-13), or 25 (**B**; *n* = 13), 50 (**C**; *n* = 13) 75 (**D**; *n* = 13) 100 (**E**; *n* = 12), or 125 (**F**; *n* = 10) µg/kg fentanyl. Grey shaded area represents the duration of IV injection and dashed line indicates control (baseline) values. Note that saline injections had no effect on V_T_, F_B_ or ${\dot{V}}_{I}$ over 15 min post-injection (*p* > 0.05), whereas there were significant (*p* < 0.05) changes in each variable following doses of fentanyl. Mixed-effects Model (REML) with Dunnett’s Multiple Comparisons (Time and Dose as factors). Interaction terms for ${\dot{V}}_{I}$ (*p* = 0.004), F_B_ (*p* = 0.0028) and V_T_ (*p* = 0.002) indicates effects of Dose and Time. Note that where multiple pairwise comparisons in which there was significant effects (*) have been omitted from the figure for clarity. However, the following timepoints were different from baseline for a given dose: 25 μg/kg (V_T_; min 2–12, 13.5, 14; F_B_; min 1–2; ${\dot{V}}_{I}$ ; min 2.5, 3, 4–7, 8–10.5, 13.5); 50 μg/kg (V_T_; min 3–8; F_B_; min 1–2; ${\dot{V}}_{I}$ ; min 10.5); 75 μg/kg (V_T_; min 2.5–4.5; F_B_; ns; ${\dot{V}}_{I}$ ; ns); 100 μg/kg (V_T_; min 1.5–6, 5.5, 6; F_B_; min 5.5; ${\dot{V}}_{I}$ ; ns), and 125 μg/kg (V_T_; ns; F_B_; ns; ${\dot{V}}_{I}$ ; min 10.5–11).

Due to the transient nature of the effects, breathing metrics were calculated in 1 s intervals, binned (30 s intervals) and normalized to each animals’ respective baseline (control; averaged across 5 min) value (Figures 3A–F). Across all animals, IV saline had no significant effect on ${\dot{V}}_{I}$ (*p* ≥ 0.4365), V_T_ (*p* ≥ 0.3875) or F_B_ (*p* ≥ 0.3018) up to 15 min post-injection (Figure 3A). However, F_B_ transiently decreased to the greatest degree during and immediately following completion of the IV fentanyl injections (∼2–5 min; *p* < 0.05 for 25 and 50 μg/kg) which led to a corresponding transient decrease in ${\dot{V}}_{I}$ at a dose of 25 μg/kg (Figure 3B; *p* < 0.05). However, fentanyl also increased V_T_ which was largely sustained up to 15 min post-injection at doses of 25–100 μg/kg (Figures 3B–E; *p* < 0.05; not significant with 125 μg/kg). The magnitude and duration of the increased V_T_ was greater than changes in F_B_ such that ${\dot{V}}_{I}$ was above control across this timeframe at all doses of fentanyl (*p* < 0.05 for 25, 50 and 125 μg/kg). Thus, the overall initial (15 min post-injection) effect of IV fentanyl at the doses tested was a transient hypopnea but sustained hyperpnea driven by increased V_T_.

**FIGURE 4:**
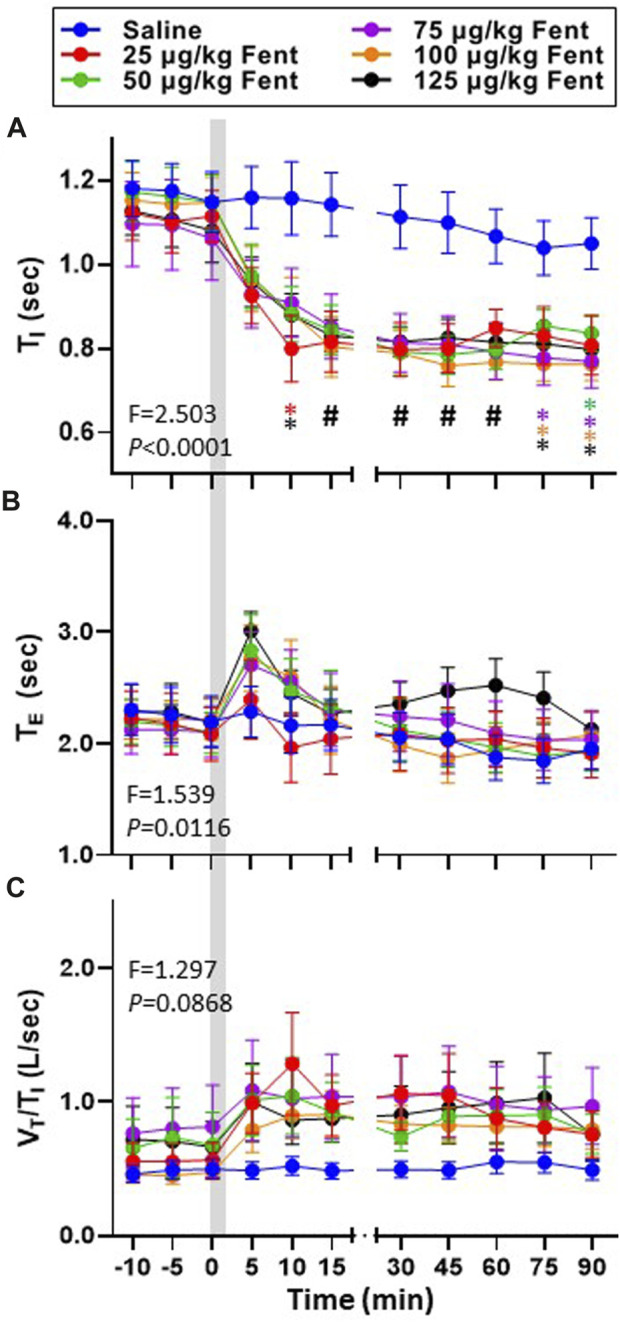
Effects of fentanyl on breath cycle timing and ventilatory drive. Inspiratory time (T_I_ (sec); **(A)**, expiratory time (T_E_ (sec); **(B)** and an index of ventilatory drive (V_T_/T_I_ (L/breath/sec)); **(C)** before and up to 90 min post-injection of IV saline (blue) or 25 (red), 50 (green), 75 (purple), 100 (orange) or 125 (black) µg/kg fentanyl. Two-way RM ANOVA (Time and Dose factors; interaction terms in figure) #*p* < 0.05 all doses vs. saline; *p < 0.05 individual dose vs. saline (n = 10–13). Grey bar indicated injection (2 min). The figure shows that fentanyl decreased inspiratory time and increased expiratory time and at most doses and increased ventilatory drive.

**FIGURE 5:**
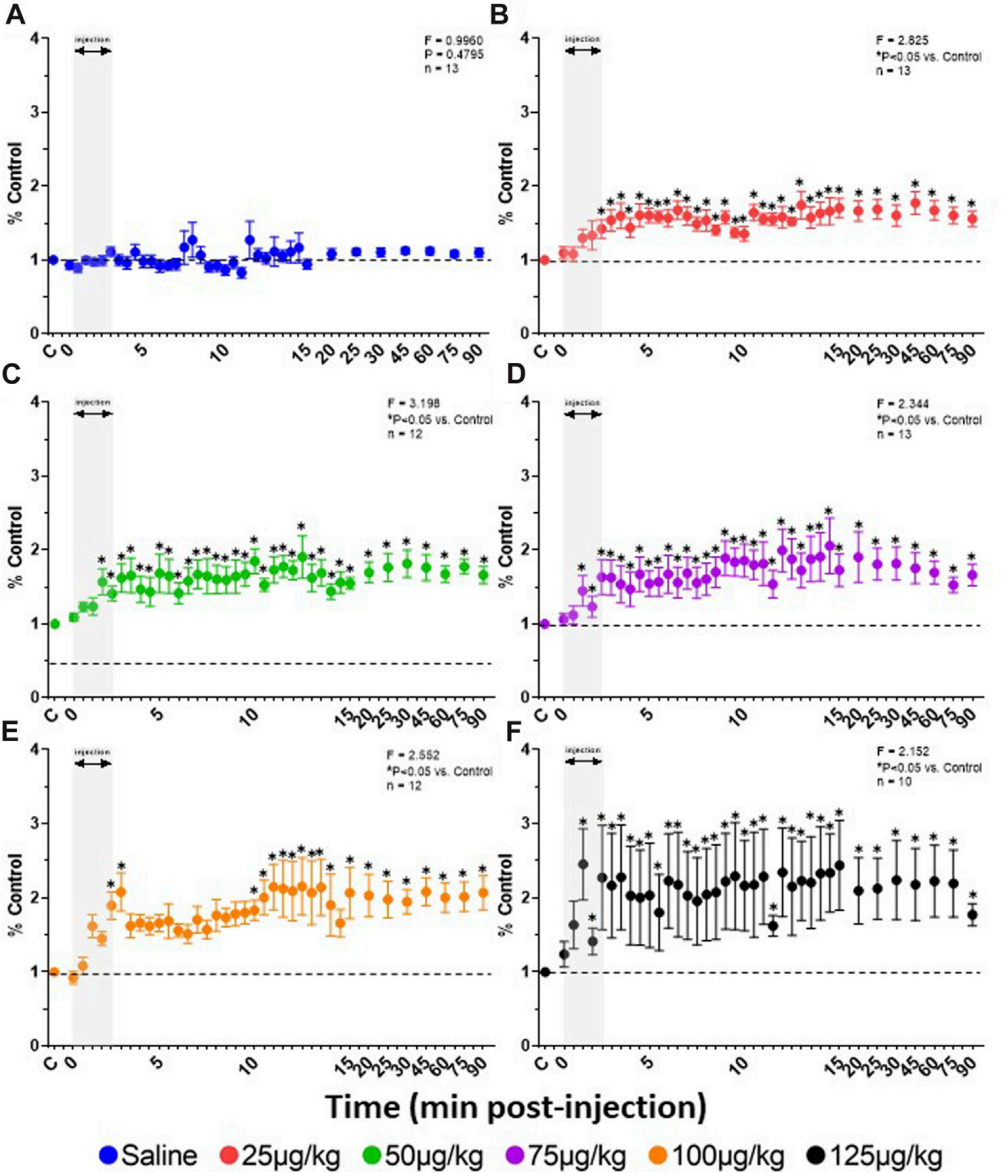
Effect of fentanyl on diaphragmatic activation. Rate of rise (mV/sec) calculations from the integrated DIA EMG (expressed as a percent of control (baseline)) during and up to 90 min after IV injections of saline (blue; **(A)** or 25 (red; **(B)**, 50 (green; **(C)**, 75 (purple; **(D)**, 100 (orange; **(E)** or 125 (black; **(F)** µg/kg fentanyl. Grey indicates injection period and dashed line control (baseline) values. Two-way RM ANOVA (Time and Dose factors; interaction terms included in figure) and Dunnett’s *post hoc*. * indicates *p* < 0.05 vs. control. This figure shows that all doses of fentanyl increased rate of rise of the diaphragm EMG indicating an excitatory effect during 90 min after the injection.

Inspiratory and expiratory time (T_I_ and T_E_, respectively) and the ratio of V_T_/T_I_ (an index of respiratory drive) was calculated during baseline conditions and for up to 90 min post-injection (Figures 4A–C). T_I_ was ∼1.1 s during baseline conditions for all goats and remained >1.0 s for up to 90 min following IV saline (Figure 4A) but decreased in a dose-independent manner after IV fentanyl such that by 30–90 min post-injection T_I_ was reduced at all doses tested (*p* ≤ 0.046). Consistent with the transient decrease in F_B_, T_E_ increased slightly within the first 5 min post-fentanyl injection with higher doses but then returned to baseline levels thereafter (Figure 4B) similar to the effects on ventilatory drive (Figure 4C). The decreased T_I_ and increased ${\dot{V}}_{I}$ (and tendency for increased ventilatory drive) suggests that diaphragmatic (DIA) activation may also be increased. Analysis of integrated DIA EMG activity to calculate the rate of rise (expressed relative to baseline) similarly was increased by the end of the IV fentanyl injection and remained elevated for up to 90 min post-injection with no effect of IV saline (Figures 5A–F). A similar integration analysis to calculate the area under the curve (AUC) on four additional accessory respiratory muscles, including the intercostal, abdominal and two upper airway (UAW1 = genioglossus; UAW2 = thyropharyngeus) muscles. Expressing AUC after injection relative to baseline, we found no change with saline injection across 90 min post-injection. However, we found increases in tonic activation in each of the four muscles with some fentanyl doses and at various timepoints after injection. UAW1 activity increased within 3–5 min following fentanyl injections at doses >25 μg/kg and remained increased up to 30 min post-injection (Supplementary Figure S5A). Similarly, transversus abdominal activity was also increased immediately following fentanyl injections at doses >50 μg/kg and remained increased for 30 min post-injection (Supplementary Figure S5B). We noted similar activation trends in both the UAW2 (Supplementary Figure S5C) and intercostal muscles (Supplementary Figure S5D), suggesting that in addition to the activation of DIA activity many accessory respiratory muscles increase in tonic activity following IV fentanyl in this dose range.

**FIGURE 6:**
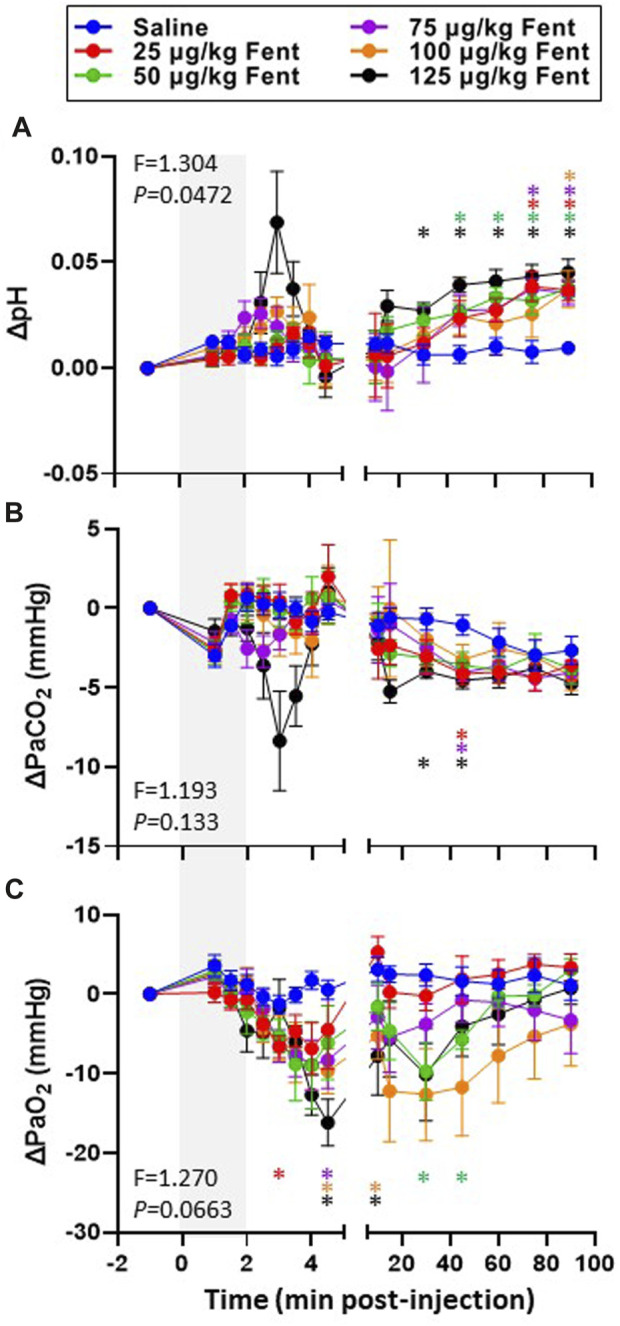
Changes in arterial blood gases with IV fentanyl injections. Shown are the changes from baseline in arterial pH **(A**; pH units), PaCO_2_ (mmHg; **(B)** and PaO_2_ **(C**; mmHg) before, during and after IV injections of saline (blue) or 25 (red), 50 (green), 75 (purple), 100 (orange) or 125 (black) µg/kg fentanyl. Mixed-effects Model (REML) with Dunnett’s Multiple Comparisons (Time and Dose as factors; Interaction terms shown in figure). * indicates *p* < 0.05 vs. saline. Note that injection of saline did not alter blood gases and pH whereas fentanyl caused transient hypercapnia and acidosis initially but alkalosis and hypocapnia over most of the post injection period. In addition, fentanyl decreased PaO_2_ for most of the post injection period.

**FIGURE 7:**
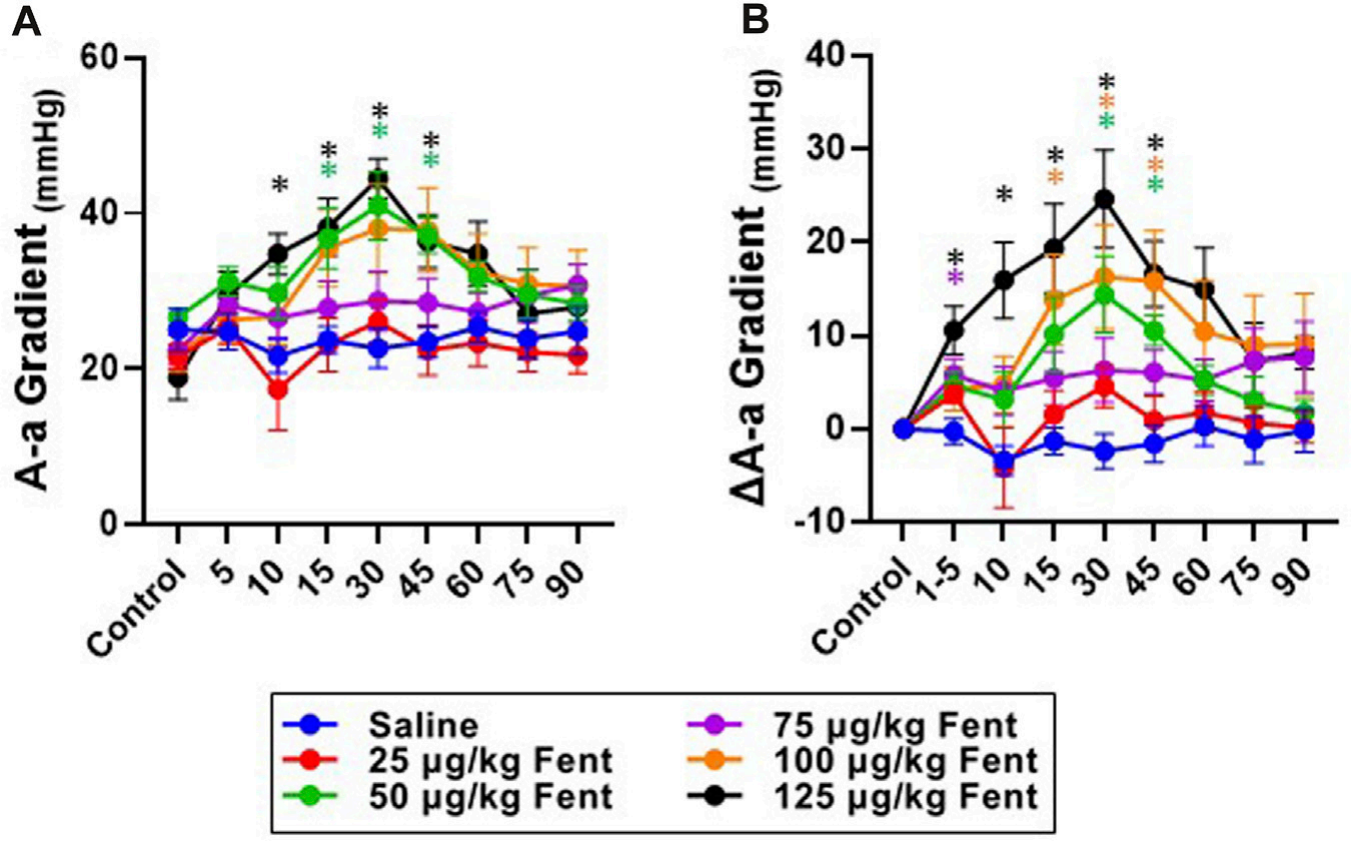
Absolute and changes in the alveolar to arterial (A–A) O_2_ gradient with fentanyl. Absolute values **(A)** or the change from baseline values (delta; **(B)** of A-a O_2_ gradients during control (baseline) conditions for up to 90 min post IV injection of saline (blue; *n* = 9) or 25 (red; n = 9), 50 (green; n = 11), 75 (purple; *n* = 13), 100 (orange; n = 12) or 125 (black; *n* = 8) µg/kg fentanyl. Mixed-effects Model (REML) with Dunnett’s Multiple Comparisons (Time and Dose as factors). *indicates *p* < 0.05 vs. saline for individual dose. Note that there was a dose- and time-dependent fentanyl-induced increase in the A-a O_2_ difference.

Arterial blood gases measured during baseline conditions were consistent across animals and did not vary between studies over the ∼3 weeks required to complete the sum of all experiments (Supplementary Table S1) indicating no lasting effects of the injections. Assessments of arterial pH, PCO_2_ and PO_2_ were made during the injection period and immediately following (1–4.5 min) and then at 10, 15, 30, 45, 60, 75 and 90 min post-injection. IV injections of saline had no major effects on arterial blood gases throughout the post-injection period, expressed either in absolute values (Supplementary Table S1) or as a change from baseline (Figures 6A–F). Arterial pH was unchanged with all doses of fentanyl during and immediately after injections but tended to increase with the highest dose (Figure 6A, B) but was consistently increased thereafter likely due to corresponding decreases in PaCO_2_ at the same timepoints (Figure 6C, D). Despite the observed hyperventilation, PaO_2_ levels were lower immediately after fentanyl injections and persisted for up to 45 min post-injection (Figure 6E, F). The slight hypoxemia shown in Figure 6 may contribute to the post fentanyl hyperpnea shown in Figure 3. However, any contribution would be minimal due to the small or lack of increase in carotid chemoreceptor activity and ventilatory stimulation with the minor changes from normal in oxygenation (Nielsen et al., 1985). We also noted minor fluctuations in blood electrolytes, with the most significant changes observed in blood glucose levels after low dose fentanyl persisting up to 90 min post-injection (Supplementary Table S1). Differences between alveolar (A) and arterial (a) PO_2_ increased following fentanyl injections at multiple doses, either expressed in absolute terms (Figure 7A) or as the change from baseline conditions (Figure 7B). Together these data suggest that at sub-lethal doses fentanyl led to sustained hyperventilation, and are highly suggestive of increases in pulmonary ventilation to perfusion ( $\dot{V}$ /Q) mismatch.

**FIGURE 8:**
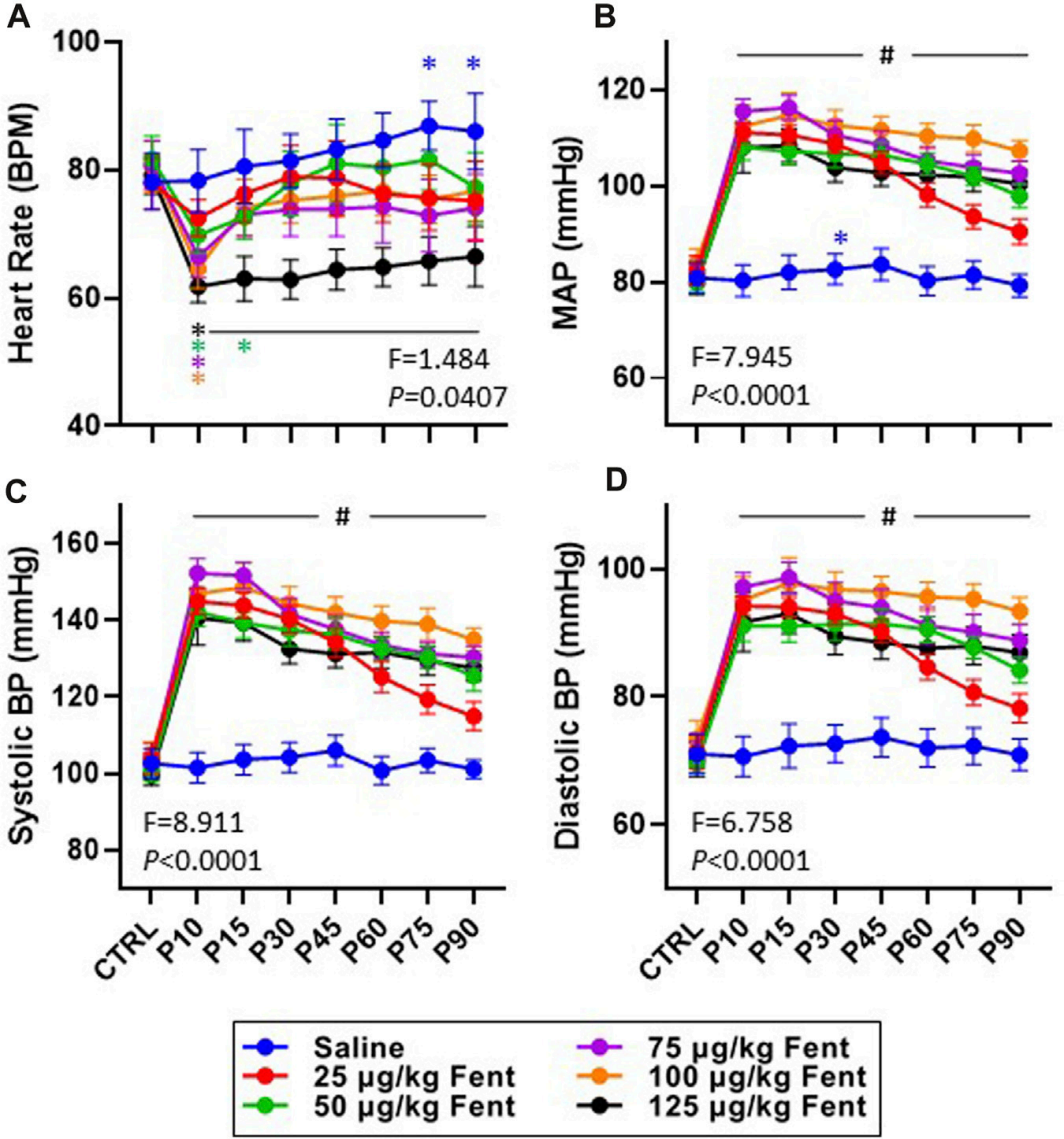
Effects of fentanyl on heart rate and blood pressures. Heart rate (beats/min; **(A)** and mean arterial (mmHg; **(B)**, systolic (mmHg; **(C)** and diastolic (mmHg; **(D)** blood pressures during control (baseline) conditions and up to 90 min post-injection (P) of saline (blue; *n* = 10) or 25 (red; *n* = 10), 50 (green; *n* = 13), 75 (purple; n = 13), 100 (orange; n = 12) or 125 (black; *n* = 10) µg/kg fentanyl. Mixed-effects Model (REML) with Dunnett’s Multiple Comparisons (Time and Dose as factors; interaction terms shown in figure). #indicates *p* < 0.05 at all doses vs. baseline; * indicates *p* < 0.05 for individual dose vs. baseline. Note also that fentanyl increased arterial blood pressure with very little time dependent recovery.

Heart rate, mean arterial blood pressure, and systolic and diastolic blood pressures were generally unchanged after IV saline injections (Figures 8A–D). However, IV fentanyl transiently decreased heart rate at lower doses but had a sustained depressant effect with 125 μg/kg fentanyl (Figure 8A). In contrast, IV fentanyl increased mean (Figure 8B), systolic (Figure 8C) and diastolic (Figure 8D) blood pressures within the first 10 min and for up to 90 min post-injection, although it appeared that these values were near baseline at the end of the study period with the 25 μg/kg dose. These effects were independent of changes in O_2_ consumption ( $\dot{V}$ O_2_) which were largely unchanged with all doses of fentanyl relative to baseline values (*p* = 0.0912) with the exception of minute 30 after 25 μg/kg (*p* = 0.306; data not shown). Expressing $\dot{V}$ O_2_ as a change from baseline conditions also suggested no change (effect of dose *p* = 0.2766; effect of dose x time *p* = 0.2917; Two-way RM ANOVA; data not shown). Lastly, although we found no differences in body temperature with all doses of fentanyl compared to saline when expressed in absolute values (*p* > 0.05; Supplementary Table S1), we found that the change in body temperature was significantly greater than saline after 25 μg/kg 10–60 min post-injection without effects of all other doses at all timepoints (*p* < 0.05; data not shown).

The effects of fentanyl on cardiovascular, metabolic rate, and body temperature responses within 90 min after injections were complicated by shifts in posture while laying and occasional shivering which varied in intensity and duration. Due to potential of affecting ventilatory responses. we did not implement methods to adjust for periods of shivering or changes in posture.

**FIGURE 9:**
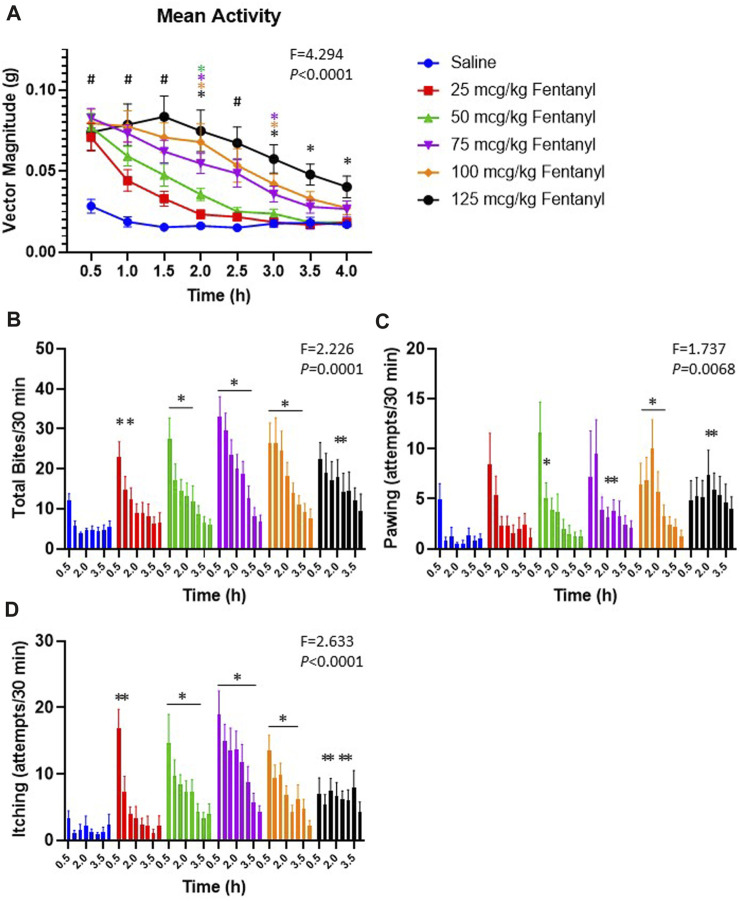
Withdrawal-like behaviors increase after fentanyl injections. Shown in **(A)** is the average bodily movement measured as vector magnitude **(G)** in 30 min intervals over 4 h beginning 1.5 h post-injection (time 0.5 is from 90–120 min post-injection). Mixed Effects analysis with multiple comparisons (Factors Time and Dose; F and *p* values shown are Interaction term). # indicates differences for all doses vs. saline; * indicates difference vs. saline with a specific dose (color-coded). Shown in **(B-D)** is the occurrences of biting, pawing and itching in each 30-min interval of the 4 h observation period. * indicates difference from saline at that timepoint (*p < 0.10). Mixed Effects analysis with multiple comparisons (Factors Time and Dose; F and *p* values shown are Interaction term).

In addition to the physiological effects of sub-lethal doses of fentanyl described above, we also quantified withdrawal-like behaviors over a 4 h period beginning 90 min post-injection. The withdrawal-like behaviors quantified (in 30 min intervals) included general increases in movement in the behavioral chamber measured with a Bioharness tracking device (hyperactivity; vector magnitude; Figure 9A), and videographic observations of numbers of biting, pawing and itching (Figures 9B–D, respectively). All animals had some level of activity in the behavioral chamber following saline injections, but activity was far greater following fentanyl injections (Figure 9A). The increased activity was dose-dependent as by 3 h their overall activity was not different from control after 25 or 50 μg/kg fentanyl but remained increased up to 4 h (5.5 h post-injection) after the 125 μg/kg dose. Similarly, the occurrences of biting (Figure 9B), pawing (Figure 9C) and itching (Figure 9D) were generally increased at various timepoints across the 4 h monitoring period after fentanyl injections compared to saline. Collectively, there was a measurable increase in withdrawal-like behaviors across a 4 h monitoring period beginning 90 min post-injection.

## Discussion

With the rise in opioid-related overdose fatalities there is a growing need to better understand the integrative physiological effects of synthetic opioids like fentanyl. Here we tested the hypothesis that sublethal doses of IV fentanyl in intact, awake, adult goats would lead to significant dysfunction in ventilation, pump and upper airway muscles, and the cardiovascular system in a time- and dose-dependent manner. We further tested the hypothesis that adult goats may also experience withdrawal-like behaviors in the hours after fentanyl administration, and thus be a good model for studying acute withdrawal. The major findings of the present study were that sub-lethal doses of fentanyl had a time- and dose-dependent stimulatory effect on many physiologic functions including ventilation, pump and airway muscle activity, and arterial blood pressure in addition to increases in the alveolar-arterial O_2_ gradient. Moreover, the fentanyl injections induced behavioral excitation which continued after physiologic effects had subsided. Collectively, these data suggest that fentanyl can be stimulatory to multiple vital functions in large animals across the dose range tested.

Opioids have long been known to affect breathing in a dose-dependent manner, where the response to fentanyl in humans is typically described as “deep and slow” breathing across a clinically-relevant dose range (Bouillon et al., 2003). Whether or not patients or animals develop a hypopnea (reduced minute ventilation) or hypoventilation (increased PaCO_2_) largely depends upon how much fentanyl they receive or their “sensitivity” to the drug. As the dose of fentanyl increases, ventilatory suppression becomes more pronounced leading to the life-threatening opioid-induced respiratory depression (OIRD). However, fentanyl has also been shown to elicit additional respiratory-related dysfunction through increased upper airway (UAW) resistance, chest wall rigidity (“Wood Chest Syndrome”; WCS), and by worsening ventilation/perfusion ratios to increase the alveolar to arterial oxygen gradient (A-a O_2_ gradient). Here we tested a dose range of IV fentanyl in a large animal species sufficient to induce sedation (immobilization) without sustained obstructive or central apneas and determine if OIRD, increased airway and chest wall muscle activity, and/or an increased A-a O_2_ gradient are present.

The overall effects of fentanyl (25–125 μg/kg) on ventilation in goats was largely stimulatory. Within seconds to minutes after fentanyl injections we noted transient periods with extremely long T_E_ and suppression of F_B_. However, V_T_ immediately increased and remained higher for up to 90 min post-injection. The relative increase in V_T_ was greater than the decrease in F_B_ such that ${\dot{V}}_{I}$ was increased across the doses of fentanyl tested. This hyperpnea was indeed hyperventilation given that PaCO_2_ was reduced and that the increased ${\dot{V}}_{I}$ occurred in the absence of a significant change in VO_2_. In addition, the rate of rise of diaphragm activation, ventilatory drive (V_T_/T_I_), arterial blood pressures and integrated activity of upper airway, intercostal and abdominal muscle activities were all increased for up to 90 min post-injection of fentanyl despite the animals being sedated. These data strongly suggest that the integrated physiological response to IV fentanyl across this dose range was largely stimulatory.

Perhaps most important among the many effects of fentanyl documented herein are the effects on airway and pump muscle activity. General muscle rigidity following opioid administration has been well-documented in animals (Hamilton and Cullen, 1953; Freye and Kuschinsky, 1976; Wells et al., 1994; Elakkumanan et al., 2008; Coruh et al., 2013; Phua et al., 2017) but reports of this phenomenon in humans are less common. WCS likely is caused by increased simultaneous activation of inspiratory and expiratory chest wall muscles to reduce compliance which could impair ventilation. However, studies on patients in which the airway was bypassed (intubation or tracheostomy) suggested mechanical ventilation was effective and thus that airway compromise may contribute more to ventilatory dysfunction with opioids than previously recognized (Scamman, 1983; Abrams et al., 1996; Bennett et al., 1997). Here we showed that tonic muscle activation in the genioglossus and thyropharyngeus was increased 2 to 3-fold for up to 30 min post-injection. In addition, fentanyl-induced increases in tonic activities of the intercostal and abdominal muscles would also support the concept that chest wall compliance and/or the mechanics of pulmonary ventilation are dysfunctional and limited. Changes in body position, from standing to supine, could affect activity of pump and airway muscles and thereby have a wooden chest like effect on breathing. We also found that the frequency of swallows was reduced by ∼50%, which indicates a decrease in airway protective reflexes. Together, these data strongly suggest fentanyl-induced increases in inspiratory airway resistance, a suppression of protective airway reflexes (swallowing), and potentially decreased chest wall compliance.

Fentanyl can also decrease arterial PO_2_ through impairment of O_2_ exchange in the lung, presumably due to increase $\dot{V}$ /Q mismatch (Meyer et al., 2015). For example, the high doses of the powerful opioid etorphine decreased respiratory rate (50%), O_2_ saturation (<85%), PaO_2_ (<60 mmHg) and increased the A-a O_2_ gradient (Meyer et al., 2010). However, administration of systemic agonists to 5-HT_1A_, 5-HT_4_ or 5-HT_7_ receptors in adult female goats improved PaO_2_ levels during etorphine treatment without increasing minute ventilation, suggesting these drugs corrected an opioid-induced $\dot{V}$ /Q mismatch. Here we demonstrated fentanyl-induced hyperventilation at multiple doses but also showed a dose-dependent hypoxemia and corresponding increase in the A-a O_2_ gradient, both of which had peak effects ∼30 min post-injection. The increased A-a O_2_ gradient was not due to hypoventilation, pointing to an independent effect of fentanyl on regional ventilation to perfusion ratios and shunt.

Discontinuation of opioid use leads to acute withdrawal symptoms in humans and pre-clinical rodent models, which include hyperalgesia, anxiety, hyperactivity, rearing vocalization, piloerection and itching (Kosten and Baxter, 2019; Pergolizzi et al., 2020; Ozdemir et al., 2023). Here we extended our studies to include observations of some withdrawal-like behaviors in a large animal model. During a 4 h observations period (beginning 90 min post-injection) we noted significant increases in locomotor behavior in addition to biting, itching and pawing. Each of these behaviors were greatest at the beginning of the observation period and waned by 3–4h, where the duration of the increases in each behavior was dose-dependent. These behaviors are similar to that observed in rodents and pigs following abstinence (Varshneya et al., 2019; Digranes et al., 2023) or naloxone-precipitated withdrawal from fentanyl (Uddin et al., 2021), suggesting that the adult goat may also be a useful model to study mechanisms or other unique aspects of withdrawal from fentanyl and its relevance to human withdrawal symptomatology.

Most clinical data on the use of opioids in patients report fairly uniform ventilatory responses of “slow and deep” breathing patterns. Less well recognized are reports that ventilatory and behavioral responses to fentanyl can vary appreciably among patients (Bouillon et al., 2003; Ikeda et al., 2005; Nishizawa et al., 2023). Moreover, most humans receiving opioids experience a euphoria (positive feeling), but ∼25% of patients instead experience dysphoria (strong negative feeling) accompanied by agitation (hoehe, 2007). The variation in human responses to opioids was the basis for a systematic analysis of variance in the human µ opioid receptor gene which revealed “abundant DNA sequence diversity suggesting numerous individual forms of the gene” (hoehe, 2007; Hoehe, 1988; Hoehe et al., 1988). Another feature of our data in goats was the unexpected, individual-dependent ventilatory responses to systemic fentanyl (Supplementary Figure S3). Most adult female goats respond over the first few minutes after fentanyl injection by decreasing breathing F_B_ and increasing V_T_ (Figure 2) which is representative of the average effect. However, two of the thirteen goats studied (∼15%) had highly stimulatory responses to fentanyl at multiple doses which lead to us prematurely stopping the experiment and removing the breathing valve and mask even with these relatively low doses of fentanyl. Only one of 13 goats required “rescue” with IV naloxone due to extremely low PaO_2_ (and high PaCO_2_) levels 30–40 min post-injection of 125 μg/kg fentanyl, and all other goats at all doses (64 of 65 experiments) did not require naloxone reversal. The hypoxemia observed in this goat was due to an above normal A-a O_2_ gradient without any measurable OIRD. Despite individual variation in physiological responses to fentanyl at a given dose, repeat experiments with 50 μg/kg fentanyl within each goat were highly reproducible in acute physiologic effects and 4 h behavioral studies (Supplementary Figure S4A-E) suggesting that randomization of dose order and a 48 h minimum between experiments had no measurable “desensitizing” effect. Irrespective of the mechanism leading to inter-animal variation in response to opioids, our data indicate that adult female goats have a human-like ventilatory response to systemic opioids suggesting goats are an appropriate model for mechanistic studies. This response differs in some respects to the response to opioids of other species. Comparisons between species are of value to gain insight into multiple factors such as drug distribution, metabolism, and drug interactions with other substances that affect the physiologic and behavioral responses to opioids. Accordingly, one goal of our future studies is to compare the effects of other opioid reversal substances shown to be effective or ineffective in other species.

In summary, herein we present findings at sublethal doses of fentanyl indicating there are time dependent changes in respiratory rhythm generation, wooden chest syndrome, alveolar-capillary gas exchange and withdrawal like symptoms that compromise normal physiologic functions and behavior. One fentanyl effect is transient depression of the respiratory rhythm generating mechanisms in the brain which was evident herein by the reduction in breathing frequency and increased duration of expiration only over the first few minutes after fentanyl injection. The dominate effect observed herein was increased breathing frequency and excitation of respiratory muscles for 30–90 min post-injection. A second fentanyl effect known as the “wooden chest syndrome” is simultaneous contraction of inspiratory and expiratory pump and airway muscles which begins a few minutes after fentanyl injection and is intermittent for at least 90 min after fentanyl injection. This effect was manifested herein by a delay in inspiratory airflow after diaphragm contraction indicative of airway constriction. A third fentanyl effect is reduced exchange of gas between alveoli and pulmonary capillaries. This effect is caused by a mismatch in delivery of air and blood to individual exchange units. The time course of each of these three effects differ, strongly suggesting that there are independent mechanisms for each fentanyl effect (neuro-respiratory output, tonic muscle activation and alveolar-capillary gas exchange). Fourth, impediment to normal physiologic function were withdrawal like symptoms characterized by random postural movements indicative of a prolonged excitatory state that persist for up to 3 hours after fentanyl injection. The present study is important in utilizing a design to avoid or minimize lethal fentanyl doses and thereby elucidate the distinct timing of emergence of fentanyl-induced physiologic and behavioral dysfunction. We conclude that sub-lethal doses of fentanyl elicit multiple compromised physiological functions independent of OIRD which may independently or in combination lead to severe outcomes.

## Data Availability

The original contributions presented in the study are included in the article/Supplementary Material, further inquiries can be directed to the corresponding author.
