# Supplementary figures and images for: Effects of sub-lethal doses of fentanyl on vital physiologic functions and withdrawal-like behaviors in adult goats
Source: Front Physiol. 2023 Oct 11;14:1277601. doi: 10.3389/fphys.2023.1277601 (PMC10598602; doi:10.3389/fphys.2023.1277601)

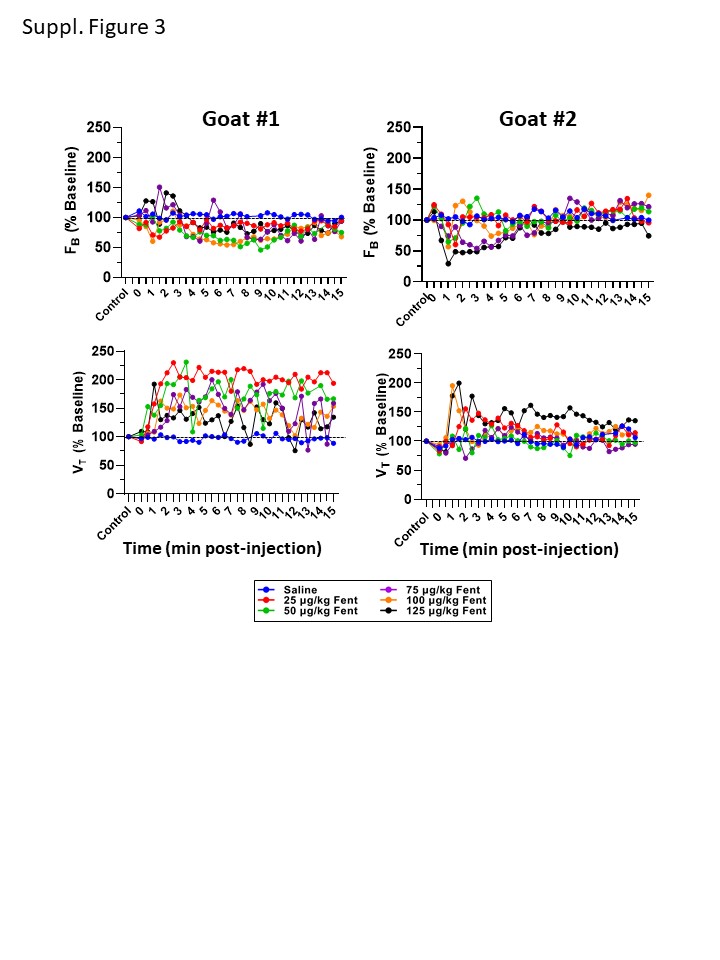

Supplement: Supplementary file 1 [file Image3.JPEG]

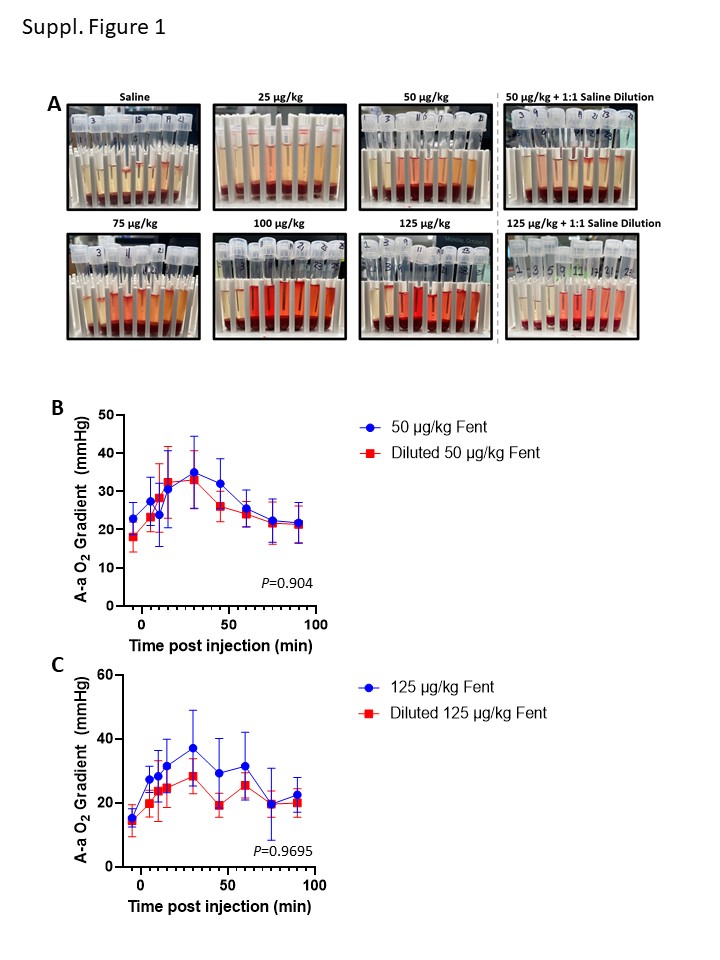

Supplement: Supplementary file 3 [file Image1.JPEG]

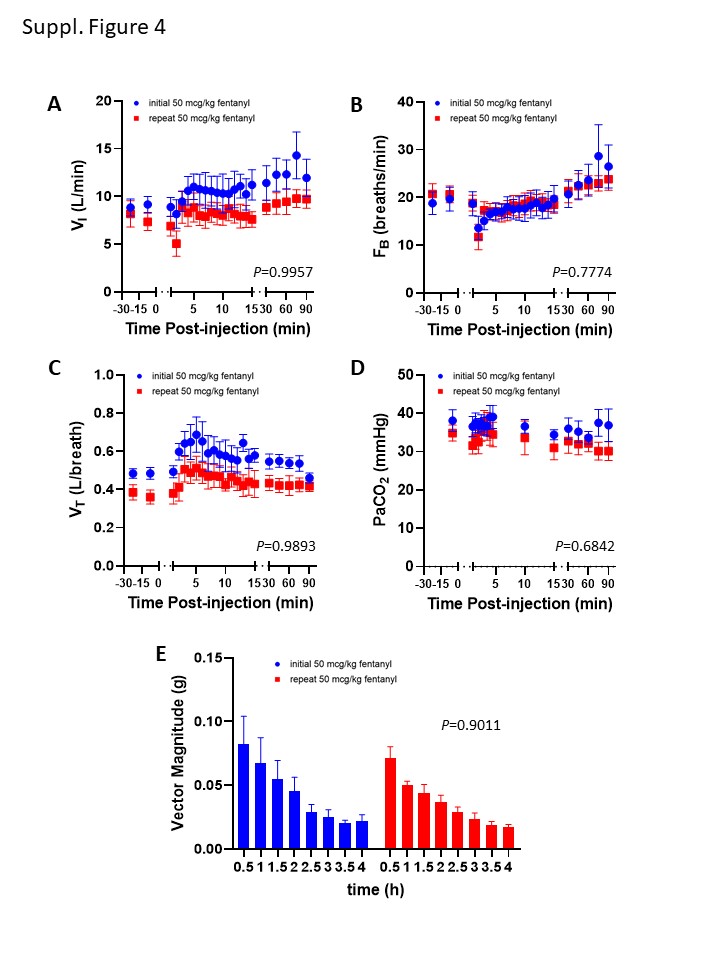

Supplement: Supplementary file 4 [file Image4.JPEG]

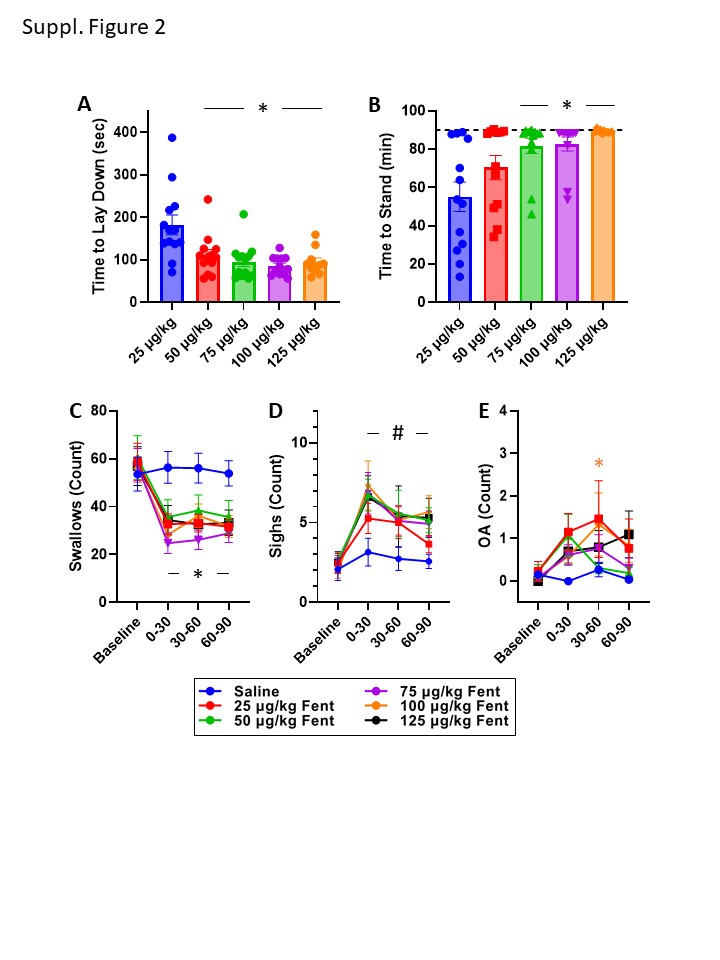

Supplement: Supplementary file 5 [file Image2.JPEG]

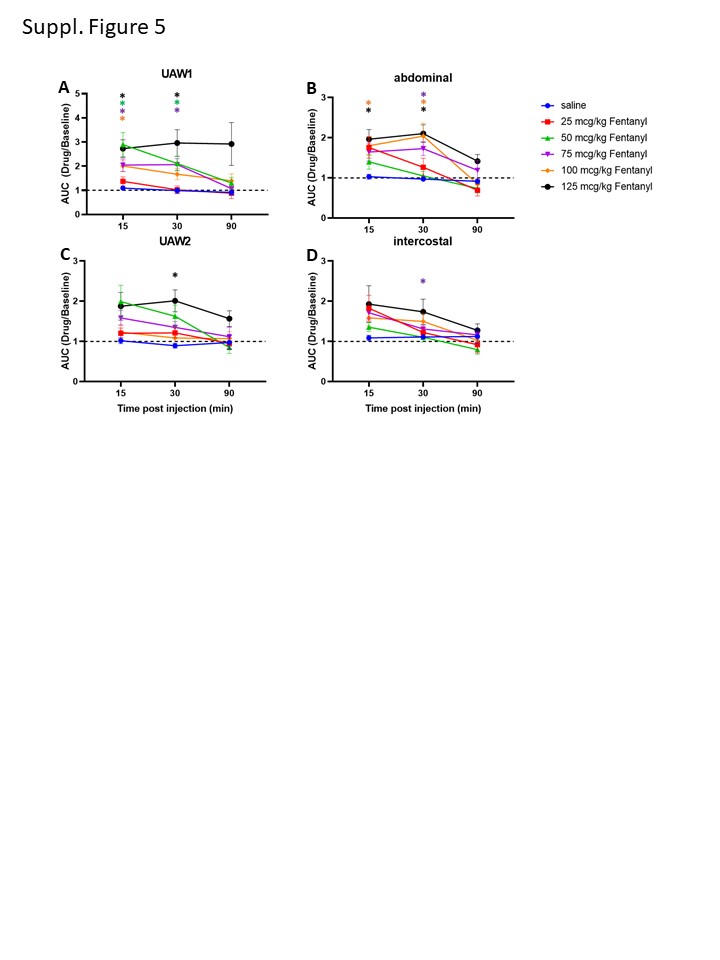

Supplement: Supplementary file 6 [file Image5.JPEG]
