# Supplementary material for: Effects of sub-lethal doses of fentanyl on vital physiologic functions and withdrawal-like behaviors in adult goats
Source: Front Physiol. 2023 Oct 11;14:1277601. doi: 10.3389/fphys.2023.1277601 (PMC10598602; doi:10.3389/fphys.2023.1277601)
Supplement: Supplementary file 2 [file Table1.pdf]

Table 1

	Time	Saline	Fentanyl (doses in µg/kg) 25	50	75	100	125	across studies
Temp (°C)	BL	39.1±0.1	39.2±0.1	39.2±0.1	39.3±0.1	39.4±0.1	39.1±0.1	ns
	15	39.2±0.1	39.5±0.1#	39.4±0.1#	39.4±0.1#	39.5±0.2	39.2±0.2	
	30	39.2±0.1	39.7±0.1#	39.6±0.2#	39.5±0.1#	39.5±0.2	39.3±0.2#	
	60	39.4±0.2#	39.9±0.1#	39.9±0.2#	39.8±0.1#	39.8±0.2#	39.4±0.2#	
	90	39.7±0.2#	39.9±0.1#	40.0±0.2#	39.8±0.1#	40.2±0.2#	39.5±0.2#	
pH (units)	BL	7.463±0.012	7.463±0.008	7.475±0.007	7.457±0.011	7.467±0.005	7.456±0.008	ns
	15	7.478±0.011#	7.469±0.024	7.493±0.007#	7.459±0.023	7.475±0.015	7.452±0.036	
	30	7.472±0.01	7.484±0.014#	7.498±0.008#	7.474±0.023	7.484±0.009	7.457±0.038	
	60	7.476±0.012	7.494±0.011#	7.513±0.009#	7.493±0.011#	7.493±0.014	7.457±0.05	
	90	7.479±0.011#	7.505±0.009#	**7.521±0.008#***	7.502±0.012#	7.506±0.011#	7.464±0.049	
$PaCO_2$ (mmHg)	BL	37.2±1.2	35.0±1.1	35.7±1.0	35.3±0.9	34.7±1.0	36.7±1.2	ns
	15	36.8±1.5	31.2±1.6#	32.9±1.0#	34.8±2.4	34.5±4.1	37.0±5.0	
	30	37.1±1.5	**29.9±1.0#***	32.6±1.1#	32.9±1.5	32.6±1.9	36.9±5.1	
	60	35.6±1.4#	29.9±1.2#	31.3±1.0#	31.7±1.1#	31.8±1.5	38.5±6.7	
	90	34.6±1.2#	30.5±1.2#	30.7±1.0#	30.3±0.9#	30.6±0.9#	35.4±4.2	
PaO_2 (mmHg)	BL	79.4±3.5	82.0±2.9	83.1±4.5	80.9±3.3	80.1±2.7	80.5±2.8	ns
	15	81.9±3.3	81.9±3.4	79.6±6.7	74.3±5.3	69.1±6.6	71.8±5.1	
	30	81.8±3.6	81.3±3.2	72.8±5.6#	75.8±3.5	68.2±6.1	65.3±5.9	
	60	80.6±3.5	82.4±3.2	81.0±3.9	78.2±3.5	72.1±5.8	72.7±5.8	
	90	79.2±3.7	83.0±1.9	85.2±4.5	79.1±4.2	74.9±4.5	78.4±4.0	
HCO_3^- (mM)	BL	26.9±1.0	25.5±1.1	26.9±1.1	25.3±0.8	25.3±0.8	26.4±1.3	ns
	15	27.6±1.2	23.6±1.3#	25.3±0.8#	24.5±0.9	25.0±1.8	24.7±1.5#	
	30	27.2±1.1	23.4±1.1#	25.3±0.8#	24.2±1.0#	24.6±1.2	25.3±1.6	
	60	26.5±1.2	23.8±1.0#	25.4±0.9	24.6±1.0	24.5±0.9	25.8±1.4	
	90	26.2±1.0	25.0±1.0	25.4±0.9	24.3±1.0	24.7±0.9	25.1±1.7	
Na^+ (mM)	BL	144.2±0.5	143.7±0.3	143.5±0.3	143.8±0.5	143.0±0.4	143.6±0.3	ns
	15	143.7±0.5#	143.1±0.5	142.3±0.3#	142.5±0.7#	**141.7±0.4#***	**141.7±0.4#***	
	30	143.3±0.5	142.3±0.5#	142.2±0.3#	142.0±0.7#	141.6±0.4#	**141.5±0.4#***	
	60	143.4±0.5#	142.3±0.6#	141.9±0.4#	141.7±0.7#	**141.5±0.4#***	141.6±0.5#	
	90	142.4±0.5#	142.0±0.5#	141.7±0.5#	141.7±0.8#	141.3±0.4#	141.8±0.4#	
K^+ (mM)	BL	4.02±0.06	3.92±0.06	3.92±0.05	3.86±0.06	3.88±0.07	3.90±0.08	ns
	15	4.12±0.07	3.99±0.07	4.04±0.07#	4.14±0.09#	4.22±0.18	4.19±0.14	
	30	4.12±0.05	4.00±0.07	3.94±0.07	3.96±0.07	3.96±0.09	3.93±0.07	
	60	4.24±0.08	**3.84±0.07***	**3.91±0.08***	**3.92±0.07***	**3.82±0.08***	4.01±0.24	
	90	4.33±0.08#	**3.78±0.07***	**3.77±0.08***	**3.95±0.05***	**3.79±0.08***	**3.75±0.06***	
Glucose (mM/dL)	BL	63.2±1.9	61.0±1.6	60.8±0.9	63.1±1.3	65.3±2.5	62.1±1.2	ns
	15	62.8±1.9	82.8±10.9	**85.9±7.2#***	**88.3±6.9#***	**94.0±8.4#***	**121.9±17.4#***	
	30	62.2±1.6	85.5±9.6	**85.5±6.8#***	**93.3±10.7***	97.9±13.7	124.0±17.4#	
	60	64.8±2.1	89.0±11.4	**83.6±5.3#***	92.0±10.0#	98.8±13.4	110.3±15.8#	
	90	66.5±2.0	85.3±9.9	**82.8±5.4#***	94.6±10.5#	96.2±12.9	104.1±12.9#	

Table 1. Body temperature, blood gases and other arterial measurements from before (baseline; BL) and up to 90 min after IV fentanyl injections. All data are mean ± SEM (n=10-13). Mixed-effects Model (REML) with Dunnett's multiple comparisons test. #indicates P<0.05 vs. BL; *(bold) indicates P<0.05 compared to saline at same timepoint; ns = no significant difference in BL values across studies.
